# Phytochemistry and Biological Activities of *Amburana cearensis* (Allemão) ACSm

**DOI:** 10.3390/molecules27020505

**Published:** 2022-01-14

**Authors:** Zildene de Sousa Silveira, Nair Silva Macêdo, Suieny Rodrigues Bezerra, Abolghasem Siyadatpanah, Henrique Douglas Melo Coutinho, Zahra Seifi, Bonglee Kim, Francisco Assis Bezerra da Cunha, Valdir de Queiroz Balbino

**Affiliations:** 1 Laboratory of Semi-Arid Bioprospecting (LABSEMA), Regional University of Cariri—URCA, Crato 63105-000, Brazil; zildenesousa15@gmail.com (Z.d.S.S.); naiirmacedo@gmail.com (N.S.M.); annesofiacrato@gmail.com (S.R.B.); cunha.urca@gmail.com (F.A.B.d.C.); 2 Graduate Program in Biological Sciences—PPGCB, Federal University of Pernambuco—UFPE, Recife 50670-901, Brazil; valdir.balbino@ufpe.br; 3 Ferdows School of Paramedical and Health, Birjand University of Medical Sciences, Birjand 97178-53577, Iran; 4 Laboratory of Microbiology and Molecular Biology (LMBM), Regional University of Cariri—URCA, Crato 63105-000, Brazil; 5 Laboratory Sciences Research Center, Golestan University of Medical Sciences, Gorgan 49189-36316, Iran; zhrseifi@gmail.com; 6 Department of Pathology, College of Korean Medicine, Kyung Hee University, Seoul 02447, Korea; 7 Korean Medicine-Based Drug Repositioning Cancer Research Center, College of Korean Medicine, Kyung Hee University, Seoul 02447, Korea

**Keywords:** *Amburana cearensis*, ethnopharmacology, phytochemistry, pharmacology, coumarins

## Abstract

*Amburana cearensis* (Allemão) ACSm. belongs to the Fabaceae family and occurs in the Brazilian semiarid, Argentina, Paraguay, Bolivia, and Peru. Numerous studies that portray its ethnobotany, use in popular medicine, chemical composition, and biological activities exist in the literature. This review aimed to provide an overview of the chemical composition, ethnopharmacology, and biological activities associated with *A. cearensis* and its isolated constituents. Information was collected from internet searches in the Scopus, Medline, PubMed, Google Scholar, and ScienceDirect databases were performed covering publications from 1997–2020. An ethnopharmacological literature analysis revealed that *A. cearensis* is used to treat a wide range of respiratory disorders in addition to intestinal, circulatory, and inflammatory problems. Coumarins, flavonoids, phenolic glycosides, phenolic acids, phenylpropanoid derivatives, and triterpenoids, among others, have been reported as active compounds, with High-Performance Liquid Chromatography (HPLC) being the main analytical technique used. The *A. cearensis* extracts and compounds presented several biological activities, including antimicrobial, antinociceptive, anti-inflammatory, antioxidant, neuroprotective, and myorelaxant activities, among others. This review provides a useful bibliography for future investigations and *A. cearensis* applications; however, future studies should focus on its toxic effects and the mechanisms of action of its extracts and isolated constituents to guide clinical applications.

## 1. Introduction

Since the beginning of human civilization, natural products such as plants have been used to treat and cure various diseases [1]. According to data from the World Health Organization (WHO), roughly 80% of the population in developing countries use phytotherapics to meet needs associated with primary health care [2]. Thus, investment in research that focuses on investigating the bioactivity of phytotherapic products has grown in recent decades. The majority of the interest lies within the field of active constituents, which are present in the composition of these products and can be effective sources for the development of bioactive derivatives with less side effects, lower costs, and thus greater accessibility [3]. Fabaceae is within the families of plants used in popular medicine. Fabaceae is also within the largest families of angiosperms, second only to Orchidaceae and Asteraceae, encompassing over 700 genera and roughly 20,000 species that are distributed worldwide [4]. In addition, Fabaceae is the second largest family, with approximately 490 species that are used in traditional medicine [5]. *Amburana cearensis* (Allemão) ACSm., popularly known as “imburana-de-odor”, “cumaru”, and “cerejeira”, among other names, is a plant species belonging to the Fabaceae family (Leguminoseae, Papilionoideae) that is well distributed in the semiarid regions of Brazil [6]. Different parts of this plant, such as the stem bark, leaves, and seeds, are widely used in popular medicine in the form of teas, infusions, and decoctions for the treatment of respiratory system disorders, such as asthma, sinusitis, bronchitis, influenza, and other purposes [7,8].

Despite the numerous biological activities that have been attributed to *A. cearensis* in the literature, a review of the plant’s bioactivities does not yet exist. Therefore, the present study aimed to systematically review the phytochemical composition, uses in traditional medicine, and bioactivities associated with *A. cearensis* as well as with some of its isolated compounds.

## 2. Materials and Methods

### 2.1. Literature Search Strategy

The name of the plant was verified using the websites www.theplantlist.org (The Plant List, 2013) (accessed on 30 November 2021) and www.ipni.org (International Plant Name Index) (accessed on 30 November 2021), with synonyms with three levels of confidence being considered. For data collection, a comprehensive search of scientific articles was performed using the following databases: Scopus, Medline, PubMed, Google Scholar, and ScienceDirect, using the following descriptors: “*Amburana cearensis*; *Amburana cearensis* and activities” in addition to its synonymous names *Torresea cearensis* and *Amburana claudii*, which have also been used to refer to this species but are not currently accepted names. Studies published from 1997 up to March 2020 were reviewed. Full-text articles were selected if the title, abstract, or keywords included the aforementioned descriptors.

### 2.2. Exclusion and Inclusion Criteria

Studies excluded from the review included ref. [1] studies that did not fall under the original study category (letters to the editor, prefaces, comments, editorials, reviews, books, book chapters, theses, and dissertations); ref. [2] repeated studies; and [3] case reports. Articles were selected based on their title, abstract, and keywords. Lastly, full articles were analyzed according to the following criteria: ref. [1] botanical aspects; ref. [2] phytochemistry; ref. [3] ethnopharmacological uses; ref. [4] bioactivities; and [5] bioactivities of isolated *A. cearensis* constituents.

## 3. Results and Discussion

Eventually, a total of 5214 studies were obtained after searching the databases. Following the selection steps based on the established inclusion and exclusion criteria, 70 articles were selected for data extraction and for the interpretation of their results. The analyzed data are discussed in the sections below.

### 3.1. Botanical Aspects

*Amburana cearensis* (Allemão) ACSm. is an arboreal species from the Fabaceae family (Leguminoseae, Papilionoideae) that is typical to open and deciduous tropical forests and is commonly found in Brazilian caatinga and cerrado regions. However, reports of the presence of this genus also exist in northern Argentina, Paraguay, Bolivia, and southeastern Peru [9,10]. According to “The Plant List” (www.theplantlist.org) (accessed on 30 November 2021), the *Amburana* genus has two species, *Amburana cearensis* (Allemão) ACSm and *Amburana acreana* (Ducke) ACSm. *A. cearensis* is characterized by its upright, brown colored stems that can reach 10 to 12 m in height [9]. *A. cearensis* has unique fruits, including Papilionoideae, that vary from green with yellowish spots to dark brown spots when dry has a wrinkled and dehiscent aspect, with winged seeds that are inside a thin membranous endocarp envelope and have an anemochory dispersion [11,12]. *A. cearensis* seeds are stenospermic, with a shape that varies between elliptical, oblong, and ovoid, with an average biometry of 14.4 mm in height, 10.4 mm in width, and 4.7 mm in thickness. *A. cearensis* seed integuments have a woody texture with a brown and opaque color and a very visible hilum that is elliptical in shape [13,14]. Its leaves are composed of 7 to 15 leaflets, which are elliptical to oval in shape, with an obtuse apex and rounded base. The inflorescences are between 2 and 5 cm long [6].

### 3.2. Uses in Popular Medicine

Traditional plants present a huge diversity of phytocompounds with possible applications against several diseases due the possible discovery of many medicines for clinical use [15]. Traditionally, *Amburana cearensis* is used by the quilombola and indigenous communities in Bolivia and northeastern Brazil for medicinal purposes [16,17,18]. The Chacobo Bolivian ethnic group (Department of Beni), who live in an endemic malaria region, use an aqueous decoction from the *A. cearensis* bark to combat fevers [19], whereas the *Tocana* ethnic group (Department of La Paz, Bolivia) uses the bark in the form of decoctions to treat abdominal and kidney pain and in the form of baths to treat rickets.

*A. cearensis* has extensive usage in popular medicine, where its bark, seeds, and fruits have been used, especially by populations in the semiarid regions of Brazil, for the treatment of respiratory system diseases such as bronchitis, asthma, rhinitis, flu, cough, and sinusitis and as an expectorant in the forms of a decoction, oral ingestion, and gargle [8,20,21,22]. The plant is also highly indicated for intestinal problems, colic, diarrhea, placental delivery, headaches, whooping cough, thrombosis, hypertension, heartburn, skin ulcers, urinary infections, dehydration, emesis, joint pain, vertigo, and fever and has been used as a depurative and diuretic to treat kidney infections as well as an antispasmodic, cicatrizing agent, tonic, and anti-anemic for fungal dermatitis, and as an antiophidic in the event of snake bites and sores [23,24,25,26,27].

Reports on the use of its bark together with other medicinal herbs in the form of syrups and macerations against fatigue and as an expectorant can also be found in the literature [28,29]. An immersion of bark in water is used in the form of baths to combat skin, throat, and gynecological inflammations [30]. The literature also reports the use of the bark and seed oil against parasitic worms, general pain, circulation problems, and wound healing [7].

### 3.3. Phytochemistry

Phytochemical investigations are important for the discovery of new bioactive phytocompounds and for the exploration of a plant’s compounds for the treatment of diseases [31]. The phytochemical characterization of different *A. cearensis* parts has been carried out by several groups in recent decades by using different analytical techniques. The isolated chemical components are mainly coumarins, flavonoids, phenolic acids, glycosylated phenols, phenylpropanoid derivatives, and triterpenoids. A summary of the phytochemical data is shown in Table 1.

#### 3.3.1. Stem Bark

Chemical composition analysis of *A. cearensis* bark extracts resulted in the isolation of coumarin and amburoside A and B [19]. Negri et al. [32] identified coumarins such as dihydrocoumarin and scopoletin; the methyl phenylpropanoid trans-3,3-dimethoxy-cinnamate; the benzoic acid methyl 3-methoxy-4-hydroxy benzoate; simple phenols such as catechol and guaiacol and the anthraquinone chrysophanol in addition to the triterpenoids lupeol and amyrin; and the steroid γ-sitosterol and the methyl ester methyl palmitate.

In the phytochemical analysis of the ethanolic extract of the bark of *A. cearensis* performed by Canuto and Silveira [33], the presence of several compounds was identified, such as coumarin; the phenolic acids vanillic acid and protocatechuic acid; flavonoids afrormosin, isokaempferide, kaempferol, quercetin, and 4′-methoxy-phiseti; and the phenolic glycoside amburoside A and a mixture of glycosylated β-sitosterol and stigmasterol. Canuto et al. [34] identified amburoside A and six new amburosides (C-H): 6-coumaryl protocatechuate, 6-hydroxycoumarin, isokaempferide, formononetin, vanillic acid, and (E)-o-coumaric acid. Leal et al. [35] also identified protocatechuic and vanillic acids in addition to coumarin and amburoside A. Sá et al. [36] showed the presence of 4-methoxy-3-methylphenol and tricyclene as well as the terpenoids α-pinene, β-pinene, and 4-hydroxybenzoic acid.

#### 3.3.2. Seeds

Costa et al. [37] reported the presence of coumarins, and l-ascorbic acid and gallic acid, ellagic acid, catechin, rutin, naringin, myricetin, and morin have also been identified [38].

The investigation of the metabolic profile of the *A. cearensis* seed extract performed by Pereira et al. [39] identified coumarin and 3-methyl-coumarin; fatty acid esters with methyl hexadecanoate, 9-cis-11-trans–octadecadienoate, methyl 13-trans-methyl-octadecanoate, and 9,12-cis-9-ethyl octadecenoate; the fatty acids hexadecanoate acetate, octadecanoate acetate, and ett octadecanoate; the steroids γ-sitosterol, campesterol, and stigmasterol; and the triterpenoid β-amyrin.

Analysis of the seed oil fatty acid profile revealed the presence of oleic (50.1%), palmitic (15.7%), linoleic (9.7%), stearic (5.3%), linolenic (5.0%), behenic (3.9%), arachidonic (2.4%), lignoceric (2.2%), lauric (0.6%), myristic (0.3%), and margarine (0.2%) [40].

#### 3.3.3. Leaves

Gouveia et al. [41] identified the presence of phenolic compounds such as protocatechuic acid, epicatechin, *p*-coumaric acid, gallic acid, and kaempferol in *A. cearensis* leaf ethanolic extract through HPLC analysis.

#### 3.3.4. Resin

Oliveira et al. [42] analyzed the *A. cearensis* resin, characterizing its constituents as follows: dilmin, lulin, erycibenin D, 7,8,3′-trihydroxy-4′-methoxyisoflavone, 7,8,3′-trihydroxy-6,4′-dimethoxyisoflavone, 3′-dihydroxy-8,4′-dimethoxyisoflavone, calycosin, odoratin, butein, cladrastrin, pratensein, 3′,4′-dimethoxy-1′-(7-methoxy-4-oxo-4H-chromen-3-yl)benzo-2′,5′-quinone, isoliquiritigenin, and homobutein.

#### 3.3.5. Cultivated Young Plants

Canuto et al. [43] investigated the phytochemical profile of cultivated young *A. cearensis* specimens, where coumarins, isokaempferidium, amburoside A and B, vanillic acid, *p*-hydroxybenzoic and aiapin acid, glycosylated (*E/Z*)-o-coumaric acid, and protocatechuic acid were identified in the ethanolic extract from young plants. Leal et al. [35] also detected vanillic acid and coumarins as constituents of the aerial parts in their study.

**Table 1:** Chemical compounds identified in *A. Cearensis* by differents analytical techniques.

Part Used	Solvents	Analytical Technique	Compounds	Citations
Stem bark	Petrol, CH_2_Cl_2_, and ethyl acetate	VLC	Coumarin and amburoside A and B	[19]
	Hexane, chloroform, ethyl acetate, acetone, and methanol	GC/EIMS	Dihydrocumarin; scopoletin; *trans*-methyl-3,4-dimethoxy cinnamate; methyl-3-methoxy-4-hydroxy benzoate; cathecol; guaiacol; chrysophanol; lupeol; amyrins; γ-sitosterol; methyl palmitate	[32]
	Ethanol	HPLC	Coumarin; vanillic acid and protocatechuic acid; afrormosin; isokaempferide; kaempferol; quercetin; 4′-methoxy-fisetin; amburoside A; β-sitosterol and stigmasterol	[33]
	Hexane	HPLC	Amburosides (A and C-H); 6-coumaryl protocatechuate; 6-hydroxycoumarin, isokaempferide, formononetin, vanillic acid, and (*E*)-*o*-coumaric acid	[34]
	Chloroform	GC/MS	4-methoxy-3-methylphenol; tricyclene; α-pinene; β-pinene and 4-hydroxybenzoic acid	[36]
	Ethanol	HPLC	Protocatechuic acid; vanillic acid; coumarin and amburoside A	[35]
Seeds	Hexane, dichloromethane, ethyl acetate, and ethanol	GC/MS	Coumarin, gallic acid, and l-ascorbic acid	[37]
	Ethanol, hexane, dichloromethane and ethyl acetate	HPLC	Coumarin; 3-methyl-coumarin; methyl hexadecanoate; methyl 9-*cis*-11-*trans*-octadecadienoate; methyl 13-*trans*-methyl-octadecanoate; methyl hexadecanoate; ethyl hexadecanoate; octadecanoate acetate; 9,12-*cis*-9-ethyl octadecanoate; ethyl octadecanoate; γ-sitosterol; campesterol; stigmasterol and β-amyrin	[39]
	Ethanol	HPLC	Gallic acid; catechin; rutin; ellagic acid; naringin; myricetin and morin	[38]
Leaves	Ethanol	HPLC/DAD	Protocatechuic acid; Epicatechin; *p*-coumaric acid; gallic acid; and kaempferol	[41]
Resin	Methanol	UPLC-DAD-QTOF-MS/MS)	Dilmin; lulin; erycibenin D; 7,8,3′-trihydroxy-4′-methoxyisoflavone; 7,8,3′-trihydroxy-6,4′-dimethoxyisoflavone; 3′-dihydroxy-8,4′-dimethoxyisoflavone; calycosin; odoratin; butein; cladrastrin; pratensein; 3′,4′-dimethoxy-1′-(7-methoxy-4-oxo-4H-chromen-3-yl)benzo-2′,5′-quinone; isoliquiritigenin and homobutein	[42]
Young cultivated plants	Ethanol	HPLC	p-hydroxybenzoic acid; ayapin, (*E*/*Z*)-*o*-coumaric acids coumarin and isokaempferide; vanillic acid, protocatechuic acid; amburosides A and B	[43]
	Ethanol	HPLC	Vanillic acid and coumarin	[35]

VLC: Vacuum liquid chromatography; GC/EIMS: gas chromatography with electron impact mass spectrometry; HPLC: high-performance liquid chromatography; GC/MS: gas chromatography coupled to mass spectrometry; HPLC/DAD: high-performance liquid chromatography equipped with a diode array detector; UPLC-DAD-QTOF-MS/MS): ultra-performance liquid chromatography coupled with diode array and quadrupole time-of-flight mass spectrometry.

### 3.4. Bioactivities Attributed to Amburana Cearensis

**Figure 1:**
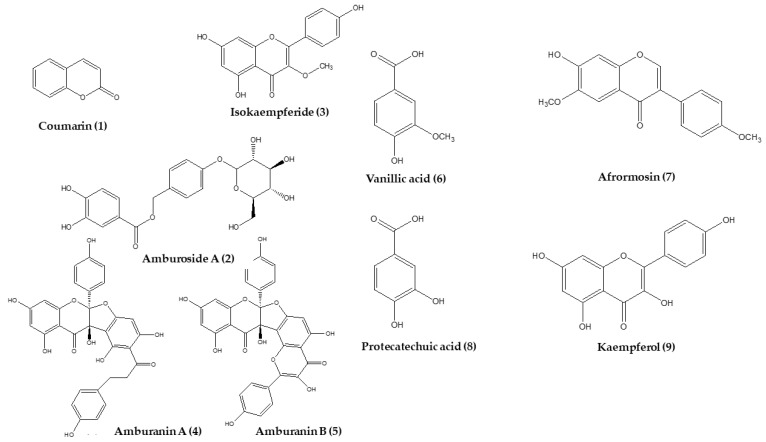
Chemical structures of the main compounds from *A. cearensis*.

Many of the medicinal properties of *A. cearensis*, such as anti-inflammatory, antinociceptive, antimicrobial, and others, have scientific support through in vivo and in vitro assays, as evidenced in this review, which justifies the application of this plant for medicinal purposes. Moreover, new evidence about other bioactivities as acaricidal, repellent, photoprotective, among others, have been observed. The major part of these studies were performed using bark extracts and fractions, with only nine compounds being evaluated for their pharmacological effects (Figure 1). The list of the ethnomedicinal scientific results available for this species are listed below. The biological properties of the crude extracts are summarized in Table 2, while their isolated phytochemicals have been compiled in Table 3.

#### 3.4.1. Antimicrobial Activity

The ethanolic extracts from the stem bark showed an antibacterial effect against *Staphylococcus epidermidis*, *S. aureus*, *S. caprie*, *S. intermedius*, *Klebsiella* spp., *Salmonella* spp., *Pseudomonas aeruginosa*, *Escherichia coli*, *Enterococcus faecalis*, *Rhodococcus equi*, *Listeria* spp., *Corynebacterium* spp., *Aeromonas* spp., *Proteus* spp, *Yersinia enterocolitica*, *Streptococcus agalactiae*, *Streptococcus suis*, *Nocardia* spp., *Vibrio* spp., *Micrococcus* spp., and *Edwadisiella* spp., with minimum bactericidal concentration (MBC) values ranging from 31.25 to 333.33 µL/mL [44].

The antibacterial activity of the leaf ethanolic extract was also demonstrated against the *S. aureus* SA-ATCC 25923 strain with a minimum inhibitory concentration (MIC) value of 512 μg/mL. Despite direct antibacterial activity against *E. coli* (MIC ≥ 1024) not being observed, a modulation of the aminoglycosides gentamicin and amikacin activity was noted, with decreases in MIC ranging from 64 to 4 μg/mL and from 128 to 8 μg/mL, respectively, against *E. coli* 27 being recorded [45].

In their study, Silva et al. [46] also found antibacterial activity for the hydroalcoholic extract against *Acinetobacter* spp., with a mean MBC of 9.375 μg/mL. The ethanolic extract, however, presented an average MBC value of 390.6 μg/mL against *Enterobacter* spp.; 1302 μg/mL against *E. coli;* and 911.4 μg/mL for the *Klebsiella* spp., genus. Similar results were found in assays performed by Peixoto et al. [47], where the *A. cearensis* bark ethanolic extract demonstrated an antibacterial potential with a mean MBC value 12,500 μg/mL against *Staphylococcus* spp. For the stem bark chloroform extract, inhibitory effects against *P. aeruginosa* and *Bacillus cereus* were also demonstrated, with MIC values of 6900 µg/mL being obtained for both strains [36].

In terms of the antibacterial activity of the seed extracts, the ethanolic extract was effective against *E. coli* (25,922), *S. aureus* (29,213), *P. aeruginosa* (27,853), *Listeria monocytogenes* (35,152), and *Shigella flexneri* (700,930), with MIC values of 1000, 250, 250, 500, and 250 μg/mL, respectively. An antibacterial effect for the seed aqueous extract against the same *E. coli*, *S. aureus*, *P. aeruginosa*, *L. monocytogenes,* and *S. flexneri* strains was also observed with MIC values of 1000, 1000, 500, 500, and 500 μg/mL, respectively [38]. Oliveira et al. [48] evaluated the antibacterial activity of the *A. cearensis* seed crude extract and its fractions (fraction 1—0 to 30%; fraction 2—30–60%; fraction 3—60–90%, of ammonium sulfate saturation). The samples did not show direct antibacterial activity against the *S. aureus* (SA10) and *E. coli* (EC06) strains, both with MIC values ≥ 1024 μg/mL. However, the *A. cearensis* seed crude extract and its fractions acted as antibiotic activity modifying agents, with a synergism being observed in the association between gentamicin and the crude extract against *E. coli* and *S. aureus*. Moreover, norfloxacin combined with the crude extract and fraction 1 also presented a significant synergism against *E. coli* and *S. aureus*. Likewise, gentamicin in combination with fractions 1 and 3 exhibited synergism against *S. aureus*. In addition, the combined effect of the crude extract and its fractions with penicillin demonstrated synergisms against *S. aureus*, while a synergism against *E. coli* was observed with the association between penicillin and the crude extract.

The isolated constituent 2-methoxy-4-methylphenol showed antibacterial activity with MIC values of 215, 215, 431, 431, 215, 215, and 215 µg/mL for *Salmonella enterica* Serotype *Typhimurium*, *E. coli*, *P. aeruginosa*, *B. cereus*, *L. monocytogenes*, *S. aureus,* and *K. pneumoniae*, respectively [36].

Many natural compounds with traditional usage have been investigated for their antimicrobial effects [49]. Regarding *A. cearensis*, its antibacterial potential is the strongest evidence of its traditional usage in treating gastrointestinal problems and infections of the urinary system [23].

The antifungal activity of the extracts and compounds isolated from *A. cearensis* is not well documented. However, the protein fraction from the seeds exhibited antifungal activity against different fungal species, presenting an inhibitory growth effect of up to 60% of the filamentous fungi *Fusarium solani*, *Fusarium oxysporum,* and *Colletotrichum lindemuthianum* at a concentration of 160 μg/mL. The 80 and 160 μg/mL concentrations also significantly inhibited the growth of *Saccharomyces cerevisiae* and *Candida albicans* yeasts, showing inhibition of up to 80% at the 160 μg/mL concentration [50]. The good effect against fungi demonstrated the traditional usage in the tretatment of fungal dermatitis [26].

Although the action mechanisms of the extracts and their compounds against bacteria have not been evidenced in the studies analyzed, these effects are possibly due to interferences in lipid bilayers inducing bacterial membrane rupture and the inhibition of processes such as biofilm formation, cell envelope synthesis, nucleic acid synthesis, electron transport chain, and ATP synthesis in these microorganisms performed by secondary metabolites such as flavonoids, which have been considered to be promising sources for antimicrobial therapy [51].

#### 3.4.2. Anti-Inflammatory, Antinociceptive and Antiedematogenic Activities

The *Torresea cearensis* (synonym of *A. cearensis*) bark hydroalcoholic extract reduced the nociception produced by acetic acid and formalin in a dose-dependent manner by 21 and 30% at the 100 and 200 mg/kg concentrations, respectively. The 200 mg/kg concentration also significantly inhibited carrageenan-induced paw edema by 30 and 48% within 3 and 4 h, respectively [52]. The reduction of the nociceptive effect induced by acetic acid indicated the effect on the peripheral analgesy mechanisms [53].

Similar results were observed by Leal et al. [54], where the oral administration of the hydroalcoholic extract at 100 and 200 mg/kg inhibited the contortions induced by acetic acid by 21 and 49%, respectively, in Wistar rats. The same concentrations also inhibited nociception by 19 and 60.1%, respectively, during the 2nd stage of the formalin test. The 200 mg/kg concentration also reduced carrageenan-induced paw edema by 48% within 4 h. Likewise, bark hydroalcoholic extract, when administered orally (400 mg/kg) or intraperitoneally (200 mg/kg), in BALB/c mice significantly inhibited antigen-induced paw edema and the production of immunoglobulin-specific ovalbumin by 50 to 80% [55]. As shown in the study by Leal et al. [56], the stem bark hydroalcoholic extract at concentrations of 200 and 400 mg/kg (administered orally) inhibited leukocyte and neutrophil migration by 37 and 42%, respectively.

According to a study by Oliveira et al. [57] the stem bark ethanolic extract demonstrated antinociceptive activity at the 200 and 400 mg/kg doses. In addition, the 100, 200, and 400 mg/kg doses decreased nociception during the 2nd stage of the formalin test by 77.5, 79.7, and 91.3%, respectively. The suppression of the pain in the second phase of the formalin assay indicated an antihyperalgesic and peripheric effect, demonstrating an anti-inflammatory effect [58].

In a comparative study using cultivated and wild *A. cearensis* species, the administration of 100–400 mg/kg of the ethanolic extract from specimens with 4, 7, or 9 months of cultivation presented antinociceptive activity, inhibiting both stages of the formalin test in mice by 32 to 64%. Similar results were observed for the bark ethanolic extract of wild species. Both extracts also reduced carrageenan-induced paw edema (Cg) at the 100–400 mg/kg concentrations [35]. Lima et al. [59] also demonstrated the antiedematogenic effects of the *A. cearensis* seed aqueous extract, where concentrations of 10 and 20% weight/volume significantly reduced carrageenan-induced paw edema in rats, with effects starting 2 h after administration of the phlogistic agent. A number of constituents isolated from *A. cearensis* also had their effects evaluated, where coumarin was one of these constituents, which at concentrations of 5–20 mg/kg, reduced, in a dose-dependent manner, the nociception produced by acetic acid and formalin [52].

Leal et al. [54] also demonstrated the antinociceptive effects of coumarin (1) in the inhibition of contortions induced by acetic acid and the formalin test at the 5, 10, and 20 mg/kg concentrations. The 10 and 20 mg/kg concentrations also inhibited carrageenan-induced paw edema in a similar manner to the aforementioned study. In the study by Leal et al. [54], orally administered coumarin (1) (20 and 40 mg/kg) significantly inhibited leukocyte and neutrophil migration in the peritoneal cavity of rats. Moreover, as demonstrated by Marinho et al. [55], coumarin (1) (10 and 20 mg/mL) reduced paw edema in animals sensitized with ovalbumin and challenged with the antigen, where coumarin, at the stated doses, induced the inhibition of anti-ovalbumin antibody titers in a similar way to dexamethasone.

Animals pretreated with amburoside A (2) (25 and 50 mg/kg) or isokaempferide (3) (12.5, 25, and 50 mg/kg) showed significant inhibition of the carrageenan-induced paw edema, where leukocyte and neutrophil migration in the peritoneal cavity of mice after the injection of carrageenan were also inhibited by the compounds [60]. The biflavonoids amburanin A and amburanin B (4–5) inhibited neutrophil degranulation by 92% and reduced human myeloperoxidase (MPO) activity by up to 53% when tested at the 25, 50, and 100 μg/mL concentrations [61].

The administration of vanillic acid (6) at the concentrations of 25 and 50 mg/kg showed antinociceptive effects by significantly reducing the two phases of the formalin test. In addition, the compound inhibited paw edema at the 25 and 50 mg/kg concentrations and leukocyte migration in the rat peritoneal cavity induced by carrageenan at the 50 mg/kg concentration [35]. The afrormosin (7) isoflavone inhibited the degranulation of the neutrophils stimulated by N-formyl-methionyl-leucyl-phenylalanine (fMLP) or phorbol 12-myristate-13-acetate (PMA), with LC_50_ values of 66.70 μM and 0.374 μM, respectively. The 3.35 and 167.6 μM concentrations inhibited myeloperoxidase activity by 39 and 76%, respectively. Cellular treatment with afrormosin at 16.76–335.2 μM concentrations reduced TNF-α release by 44% [62].

#### 3.4.3. Myorelaxant Activity

The *A. cearensis* bark hydroalcoholic extract caused concentration-dependent relaxations in the trachea of guinea pigs pretreated with 0.3 μM carbacol, obtaining an EC_50_ value of 3.64 ± 0.4 mg/mL [54]. In the study by Leal et al. [56] the same extract evoked a concentration dependent relaxation in guinea pig trachea with an IC_50_ value of 3.16 ± 0.55 mg/mL in the presence of three antagonists.

Studies also revealed the coumarin (1) myorelaxant activity in the trachea of guinea pigs pre-contracted with 0.3 μM carbacol, which obtained an EC_50_ value of 0.08 ± 0.01 mg/mL [54]. Similar results were observed by Leal et al. [56], where coumarin (1) showed a myorelaxing effect in guinea pig trachea pre-contracted with carbacol (0.3 μM), histamine (0.1 μM), or KCl (0.1 M), obtaining IC_50_ values of 0.045 ± 0.013, 0.084 ± 0.016, and 0.087 ± 0.006 mg/mL. These studies demonstrated the interaction of isolated compounds such as the coumarins and isokaempferol present in *A. cearensis* extracts, possibly conferring a myorelaxing effect to this plant.

#### 3.4.4. Antioxidant Activity

The leaf ethanolic extract as well as the ethyl acetate and methanolic fractions, showed antioxidant activity when evaluated by the DPPH assay at the 0.1 mg/mL concentration, with an antioxidant potential of 93.63, 83.21 and 93.01%, respectively. The bark ethanolic extract, on the other hand, presented an antioxidant potential of 94.55% at the 1 mg/mL concentration [50]. Antioxidant activity for the seed ethanolic extract at 30, 60, and 120 μg/mL concentrations with an IC_50_ value of 17.95 μg/mL was also observed [38].

Lopes et al. [62] evaluated the inhibitory effect of afrormosin (7) on the superoxide anion production and the total generation of reactive oxygen species (ROS) in human neutrophils stimulated by phorbol 12-myristate-13-acetate (PMA) using lucigenin-enhanced chemiluminescence assays (LucCL) and luminol (LumCL). The results showed the isoflavone-inhibited ROS production with a significantly higher inhibitory effect in the LumCL (IC_50_ = 19.09 μg/mL) than in the LucCL (IC_50_ > 100 μg/mL) assay.

Antioxidative agents have a role in inhibiting oxidative stress that is induced due to free radicals, protecting the cells. Medicinal plants present a huge variety of compounds with antioxidative properties, with this effect being mediated by different mechanisms [63]. The antioxidant potential of the Fabaceae family has been demonstrated by several studies, most of which have been associated with the occurrence of flavonoids and highlighting the isoflavone class, which are considered to be the most bioavailable flavonoids and are restricted to some legume subfamilies [51].

#### 3.4.5. Neuroprotective Activity

Several studies have shown evidence that natural products can reduce neuronal damage, making them a possible source for the development of new drugs and therapeutic strategies for the treatment of these diseases [64]. The dry extract, phenol fraction and amburoside A (2), obtained from *A. cearensis* demonstrated neuroprotection over microglial cells at the 5–200 mg/mL concentration [65]. The ethanolic, hexane, dichloromethane, and ethyl acetate extracts obtained from *A. cearensis* seeds presented a neuroprotective potential over PC12 neural cells exposed to glutamate-induced neuronal damage (1 mM) at concentrations of 0.1–1000 µg/mL [39]. The ethanol and dichloromethane extracts from the seeds also demonstrated the neuroprotective effects against the neural damage induced by glutamate in rat cerebellar cultures at a concentration of 0.1 µg/mL, with this effect being possibly associated with the increased expression of the astrocytic enzyme glutamine synthetase from the glial fibrillary acidic protein (GFAP) and β-III tubulin, indicating glial and neuronal preservation, respectively [40].

The neuroprotective effects observed are possibly due to the metabolites present in *A. cearensis* with antioxidant and anti-inflammatory properties that are able to regulate the intracellular signaling cascades of glutamate excitotoxicity, which is a critical component in the development of neurological and neurodegenerative diseases, and this regulation may minimize the main causes of neuronal death [40].

#### 3.4.6. Cytotoxic and Antiproliferative Activities

The *A. cearensis* stem powder hydroalcoholic extract presented cytotoxicity in L929 fibroblasts and human keratinocytes (HaCaT) at the 31.25, 62.5, 125, 250, 500, and 1000 μg/mL concentrations. The same concentrations also demonstrated antiproliferative activity in human breast adenocarcinoma cell lines (MDA-MB-231 and MCF7) and mouse mammary gland tumors (4T1) [66]. At a concentration of 1000 µg/mL, seed hexanic extract reduced the viability of Wistar rat cerebellar cells by 30% [40].

The compounds kaempferol (9), isokaempferide (3), amburoside A (2), and protocatechuic acid (8) showed cytotoxic activity against five tumor cell lines: B-16 cells (murine skin cancer), HCT-8 (human colon cancer), and MCF-7 (breast cancer human) as well as the EMC and HL-60 (leukemia cancer) cell lines from tumor cells. Isokaempferide obtained IC_50_ values of 2.6 μg/mL, 3.0 μg/mL, 5.4 μg/mL, 5.5 μg/mL, and 3.6 μg/mL for EMC, HL-60, HCT-8, MCF-7, and B-16, respectively. Meanwhile, the IC_50_ values for protocatechuic acid were >25, 20.7, >25, >25, and >25 μg/mL for EMC, HL-60, HCT-8, MCF-7, and B-16, respectively. Amburoside obtained an IC_50_ value >25 μg/mL for all tested strains, while kaempferol obtained an IC_50_ of 13.4, 22.7, 15.2, 21.2, 11.5 μg/mL for the lines EMC, HL-60, HCT-8, MCF-7, and B-16, respectively [67]. Based on these facts, extracts and compounds from *A. cearensis* demonstrated activity against several cancer cell lines. However, the mechanisms involved in these activities were not elucidated, making more studies necessary.

#### 3.4.7. Allelopathic Activity

Allelopathy is a defense mechanism observed on several organisms that are mainly present in plants and involves the production and liberation of bioactive compounds in the environment. On plants, these compounds affect the growth and the development of neighbouring plants [68]. Previous reports have demonstrated the allelopathic effects on the germination of *Lactuca sativa* seeds that have received aqueous and methanolic treatments at concentrations 5, 10, and 15 g/L and 1, 5, 10, and 15 g/L, respectively, of the *A. cearensis* ground seed. Both extracts also showed an allelopathic activity on the germination of *Raphanus sativus* L. seeds treated with 15 g/L of *A.* *cearensis* extract [69]. The leaf aqueous extract almost completely prevented the germination of *Amaranthus deflexus* at the 50 and 100 g/L doses, with a 99.3 and 99.5% decrease in germination percentage and germination velocity index, respectively, at the highest concentration [70].

The seed hydroalcoholic extract prevented the germination of *Cucumis melo* L. seeds and the emergence of seedlings at the 1, 0.5, and 0.25% weight/volume concentrations. However, the leaf extract evaluated in the same study did not prevent seed germination, despite the seedlings presenting abnormalities that exceeded 25% at the highest concentration [71]. Similar results were observed in the study by Oliveira et al. [48], where the hexane and dichloromethane fractions of the seed extract affected the emergence and initial growth processes of *Cucumis melo* L. at 1 and 0.5% weight/volume concentrations.

The allelochemicals belong to several different classes of secondary metabollites, including phenols, benzoic acid, and derivates of cinnamic acid, terpenoids, glicosides, among others [72]. However, the inhibition of germination in both weed seedlings and in *Amaranthus deflexus* as well as in edible plants such as *Lactuca sativa*, *Raphanus sativus* and *Cucumis melo* indicates a possible non-biosselectivity, which could result in a threat to non-target organisms. Therefore, this highlights the need for studies to systematically determine the bioselectivity and biosafety of the allelochemicals present in *A. cearensis*, which will guide the use of this species in practical applications.

#### 3.4.8. Other Bioactivities

In addition to the aforementioned bioactivities, *A. cearensis* has other properties that have been described in the literature. According to the study carried out by Trevisan et al. (2003) [73], the ethanolic extract of the stem bark caused 100% inhibition of acetylcholinesterase (AChE) enzyme activity at a concentration of 2.3 mg/mL. Meanwhile the hydroalcoholic extract obtained from the stem powder caused 83% inhibition, with an IC_50_ value of 0.3789 mg/mL [66].

The *A. cearensis* leaf ethanolic extract at a concentration of 0.2 mg/mL increased the preservation of pre-antral goat follicles for up to 6 h [41,74]. At a concentration of 0.1 mg/mL, the same extract also had a significant effect on the survival and in vitro development of secondary follicles isolated from sheep (*Ovis aries*) [75]. Similar results were observed in the study by Menezes et al. [76], where the *A. cearensis* leaf ethanolic extract at a concentration of 0.2 mg/mL maintained follicular survival and DNA integrity in a similar manner to those observed for the minimum essential medium (MEM) over 24 h.

The seed aqueous extract exhibited marked larvicidal activity against the *Aedes aegypti* mosquito, with an LC_50_ value of 8.10 ± 0.27 mg/mL within 24 h of exposure [77]. The leaf and stem bark powder (2 g) presented repellent activity against the bean weevil *Callosobruchus maculatus*, obtaining repellency rates of 0.46 ± 0.05 and 0.42 ± 0.04, respectively [78]. In addition, the hexane fraction from the leaf ethanolic extract at a concentration of 25 mg/mL showed in vitro acaricidal activity against engorged *Rhipicephalus* (*Boophilus*) *microplus* females, reaching oviposition and hatching inhibition values of 52.7% and 39.0%, respectively [79]. In the study by Lima et al. [59], the *A. cearensis* seed aqueous extract demonstrated mutagenic activity over *Allium cepa* at concentrations of 0.1 and 0.5 mg/mL.

An in vitro photoprotective activity for the *A. cearensis* leaf ethanolic extract has also been described, with UVB radiation absorption efficiency at wavelengths being in the 280 to 320 nm region, with a sun protection factor (SPF) equal to 17.60 at a concentration of 0.2 mg/mL [80].

Coumarin (1), identified as 1,2 benzopyrone, isolated from *A. cearensis* seeds at concentrations of 25, 50, 100, 250, and 500 mg/mL showed antileishmanial activity against *Leishmania chagasi* promastigotes [81].

Treatment with amburoside A (2) at 25 and 50 mg/kg doses significantly inhibited the increase in aspartate aminotransferase (AST) and alanine aminotransferase (ALT) serum levels in animals poisoned by carbon tetrachloride (CCl_4_). In liver tissues, the compound inhibited the formation of CCl_4_-induced thiobarbituric acid reactive substances (TBARS) at both doses, indicating a CCl_4_-induced lipid peroxidation blockade [82].

Although the extracts and phytocompounds isolated from *A. cearensis* present a wide range of therapeutic properties that have already supported by experimental results, some medicinal properties such as depurative, diuretic, antispasmodic, and antivenom reported for this species remain unexplored, highlighting important areas of research with the opportunity for chemical and biological exploration to carry out in vivo and in vitro assays as well as for the evaluation of the mechanisms of action of the crude extracts and isolated compounds.

**Table 2:** Biological activities of *Amburana cearensis*.

Activity Tested	Extract/Part	Model Used	Concentrations/Dosage	Citations
Antibacterial	Ethanol/stem bark	*Staphylococcus epidermidis*; *Staphylococcus aureus*; *Staphylococcus caprie*; *Staphylococcus intermedius*; *Klebsiella* spp.; *Salmonella* spp., *Pseudomonas aeruginosa*; *Escherichia coli*; *Enterococcus faecalis*; *Rhodococcus equi*; *Listeria* spp.; *Corynebacterium* spp.; *Aeromonas* spp.; *Proteus* spp.; *Yersinia enterocolitica*; *Streptococcus agalactiae*; *Streptococcus suis*; *Nocardia* spp.; *Vibrio* spp.; *Micrococcus* spp.; *Edwadisiella* spp.	MBC—125; 145.8; 333.33; 31.25; 83.33; 145.8; 166.7; 166.7; 187.5; 187.5; 125; 41.67; 62.5; 281.3; 104.2; 250; 83.33; 125; 31.25; 125; 62.5 µL/mL, respectively	[44]
	Ethanol/leaves	*S. aureus* 25,923	MIC—512 μg/mL	[45]
	Hydroalcoholic	*Acinetobacter* spp.	MBC—9.375 μg/mL.	[46]
	Ethanol	*Enterobacter* spp.; *E. Coli*; and *Klebsiella* spp.	MBC—390.6; 1.302; and 911.4 μg/mL, respectively	[46]
	Ethanol/stem bark	*Staphylococcus* spp.	MBC—12.500 μg/mL	[47]
	Chloroform/stem bark	*P. aeruginosa* and *Bacillus cereus*	MIC—6900 µg/mL	[36]
	Ethanol/seeds	*E. coli* (25,922); *S. aureus* (29,213); *P. aeruginosa* (27,853); *L. monocytogenes* (35,152); *Shigella flexneri* (700,930)	MIC: 1000; 250; 250; 500; and 250 μg/mL, respectively	[38]
	Crude and fractions (ammonium sulfate concentration: 0–30%, 30–60% and 60–90%/seeds	*S. aureus* (SA10) and *E. coli* (EC06)	MIC ≥ 1024 μg/mL	[48]
Antifungal	Proteic fractions/seeds	*Colletotrichum lindemuthianum*; *Fusarium oxysporum*; *Fusarium solani*; *Candida albicans;* and *Saccharomyces cerevisiae*	Filamentous fungi: 160 μg/mL; Yeasts: 80 and 160 μg/mL	[50]
Antinociceptive and antiedematogenic	Hydroalcoholic/stem bark	Swiss mice and Wistar rats	100 and 200 mg/kg	[52]
	Hydroalcoholic/stem bark	Swiss mice and Wistar rats	100 and 200 mg/kg, respectively	[54]
	Hydroalcoholic/stem bark	Mice BALB/c	200 mg/kg and 400 mg/kg	[55]
	Ethanol/stem bark	Swiss mice and Wistar rats	100; 200 and 400 mg/kg	[57]
	Aqueous/seeds	*Rattus norvegicus*	10 and 20% *w/v*	[59]
Anti-inflammatory	Hydroalcoholic/stem bark	Wistar rats	200 and 400 mg/kg	[56]
Myorelaxant	Hydroalcoholic/stem bark	Guinea-pig isolated trachea	EC_50_: 3.64 ± 0.4 mg/mL	[54]
	Hydroalcoholic/stem bark	Guinea-pig isolated trachea	IC_50_ 3.16 ± 0.55 mg/mL	[56]
Antioxidant	Ethanol/leaves and bark. Methanol and ethyl acetate fractions/leaves	DPPH	0.1 and 1 mg/mL	[50]
	Ethanol/seeds	DPPH	IC_50_ 17.95 μg/mL	[38]
Neuroprotective	Ethanolic, hexane, dichloromethane, and ethyl acetate/seeds	Neural PC12 cells	0.1–1000 µg/mL	[39]
	Ethanol and dichloromethane/seeds	Cerebellar cells of wistar rats	0.1 µg/mL	[40]
	Dry extract and phenol fraction	Microglial cells	5–200 mg/mL	[65]
Antiproliferative	Hydroalcoholic/stem powder	HaCaT; MDA-MB-231 and MCF7; 4T1	31.25; 62.5; 125; 250; 500; and 1000 μg/mL	[66]
	Hexane/seeds	Cerebellar cells of Wistar rats	1000 µg/mL	[40]
Allelopathic	Aqueous and metanol/seeds	*Lactuca sativa*	5, 10, and 15 g; 1; 5; 10; and 15 g, respectively	[69]
	Aqueous and methanol/seeds	*Raphanus sativus* L.	15 g	[69]
	Aqueous extract/leaves	*Amaranthus deflexus*	50 and 100 g/L	[70]
	Hydroalcoholic extract/seeds	*Cucumis melo* L.	1; 0.5; and 0.25% *w/v*	[71]
	Hydroalcoholic/leaves	*Cucumis melo* L.	1; 0.5; and 0.25% *w/v*	[71]
	Hexane and dichloromethane fracionas/seeds	*Cucumis melo* L.	1 and 0.5% *w/v*	[48]
Survival of preantral follicles	Ethanol/leaves	Ovarian cortical tissues goats	0.2 mg/ml	[41]
	Ethanol/leaves	Ovarian cortical tissues ovine (*Ovis aries*)	0.1 mg/mL	[75]
	Ethanol/leaves	Ovarian cortical tissues ovine	0.2 mg/mL	[76]
Larvicidal	Aqueous/seeds	*Aedes aegypti*	LC_50_ 8.10 ± 0. 27 mg/mL after 24 h of exposure	[77]
Repellency	Powder/leaves and bark	*Callosobruchus maculatus*	2 g	[78]
Acaricidal	Hexane fraction/leaves	*Rhipicephalus* (*Boophilus*) *microplus*	25 mg/mL	[79]
Mutagenic	Aqueous/seeds	Meristematic cells of *Allium cepa*	0.1 mg/mL and 0.5 mg/mL	[59]
Photoprotective	Ethanol/leaves	-	SPF = 17.60	[80]

HaCaT: human keratinocytes; MDA-MB-231 and MCF7: human breast adenocarcinoma; 4T1: Mouse tumor mammary gland; MIC: minimum inhibitory concentration; MBC: minimum bactericidal concentration; LC_50_: median lethal concentration; DPPH: 2,2-Diphenyl-1-picrylhydrazyl radical; SPF: protection factor.

**Table 3:** Biological activities of compounds isolated from *A. cearensis*.

Phytochemicals	Activity Tested	Model Used	Concentraction /Dosage	Citations
Coumarin (1)	Antinociceptive and antiedematogenic	Swiss mice and Wistar rats	5–20 mg/kg and 10 mg/kg	[52]
Coumarin (1)	Anti-inflammatory, antinociceptive	Swiss mice and Wistar rats	5; 10; and 20 mg/kg	[54]
Coumarin (1)	Anti-inflammatory	Wistar rats	20 and 40 mg/kg	[54]
Coumarin (1)	Anti-inflammatory and antiedematogenic	BALB/c mice	10 and 20 mg/mL	[55]
Coumarin (1)	Myorelaxant	Guinea pig trachea	EC_50_ 0.08 ± 0.01 mg/mL	[54]
Coumarin (1)	Myorelaxant	Guinea pig trachea	IC_50_ 0.045 ± 0.013; 0.084 ± 0.016 and 0.087 ± 0.006 mg/mL (carbachol, histamine and KCl)	[56]
Coumarin (1)	Antileishmanial	*Leishmania chagasi* (promastigotes)	25; 50; 100; 250; and 500 mg/mL	[81]
Amburoside A (2)	Antiedematogenic and anti-inflammatory	Swiss mice and Wistar rats	25 and 50 mg/kg	[60]
Amburoside A (2)	Neuroprotective	Rat microglial cell cultures	5-200 mg/mL	[65]
Amburoside A (2)	Hepatoprotective	CCl_4_-induced hepatotoxicity in Wistar rats	25 and 50 mg/kg	[82]
Isokaempferide (3)	Cytotoxic	CEM; HL-60; HCT-8; MCF-7 and B-16	IC_50_: 2.6 μg/mL (CEM); 3.0 μg/mL (HL-60); 5.4 μg/mL (HCT-8); 5.5 μg/mL (MCF-7); and 3.6 μg/mL (B-16)	[67]
Amburanins A and B (4 and 5)	Anti-inflammatory	Human neutrophils	25; 50 and 100 μg/mL	[61]
Vanillic acid (6)	Antinociceptive, antiedematogenic and anti-inflammatory	Swiss mice and Wistar rats	25 and 50 mg/kg	[35]
Afrormosin (7)	Anti-inflammatory	Human neutrophils stimulated by fMLP or PMA	IC_50_ 66.70 μM (fMLP) and 0.374 μM (PMA); 3.35–167.6 μM (inhibition of MPO); 16.76–335.2 μM (reduced TNF-a level)	[62]
Afrormosin (7)	Antioxidant	Human neutrophils (chemiluminescent Probes-luminol and lucigenin	CL_50__:_ Lucigenin (>100 μg/mL) and luminol (19.09 μg/mL)	[62]
Protecatechuic acid (8)	Cytotoxic	CEM; HL-60; HCT-8; MCF-7; and B-16	IC_50_: >25.0 (CEM); 20.7 (HL-60); >25.0 (HCT-8); >25.0 (MCF-7); >25.0 μg/mL (B-16)	[67]
Kaempferol (9)	Cytotoxic	CEM; HL-60; HCT-8; MCF-7; and B-16	IC_50_ 13.4 μg/mL (CEM); 22.7 μg/mL (HL-60); 15.2 μg/mL (HCT-8); 21.2 μg/mL (MCF-7); 11.5 μg/mL (B-16)	[67]
2-methoxy-4-methylphenol	Antibacterial	*Salmonella entérica*; *E. Coli*; *P. Aeruginosa*; *B. Cereus*; *L. Monocytogenes*, *S. Aureus* and *K. Pneumoniae*	MIC—215; 215; 431; 431; 215; 215; and 215 µg/mL, respectively	[36]

EC_50_: half maximal efective concentration; IC_50_: half maximal inhibitory concentration; CL_50_: median lethal concentration; B-16 (murine skin cancer); HCT-8 (human colon cancer); MCF-7 (human breast cancer); CEM and HL-60 (leukemia cancer); MPO: mieloperoxidase; fMLP: formyl-methionyl-leucyl-phenylalanine; PMA: phorbol-12-myristate-13-acetate; TNF-α: tumor necrosis factor alpha.

## 4. Conclusions

Phytochemical, ethnobiological, and pharmacological studies of *Amburana cearensis* are well reported. Many of these activitites were elucidated based on studies with different extracts and phytocompounds that had been isolated, with its traditional usages being supported by these bioactivities. For example, all of the antibacterial, antifungal, anti-inflammatory, antinociceptive, antioxidant, and myorelaxant activities studied can be directly associated with traditional usages against infections caused by bacteria and fungi, against respiratory and gastrointestinal illnesses, fever, malaria, among others. New biological activities such as acaricidal, larvicide, repellent, photoprotective, antileishmanial, activities were observed, among others. However, there are some activities that are unable to be associated with traditional usage, as depurative, diuretic, antispasmodic, antivenom, activities, among others. The extensive use of this plant suggests relative security against acute toxicity. More studies determining the general toxicity of this plant are necessary.

## Data Availability

Not applicable.

## Sample Availability

Samples of the compounds are not available from the authors.
